# Complete genome sequence of the fish pathogen *Flavobacterium psychrophilum* ATCC 49418^T^

**DOI:** 10.1186/1944-3277-10-3

**Published:** 2015-01-21

**Authors:** Anson KK Wu, Andrew M Kropinski, John S Lumsden, Brian Dixon, Janet I MacInnes

**Affiliations:** 1 Department of Pathobiology, Ontario Veterinary College, University of Guelph, 50 Stone Road East, Guelph, Ontario N1G 2W1, Canada; 2 Department of Biology, 200 University Avenue West, Waterloo, Ontario N2L 3G1, Canada

**Keywords:** Aerobic, Gram negative, Psychrotolerant, Fish pathogen, *Flavobacterium*, Bacterial cold water disease, Rainbow trout fry mortality syndrome

## Abstract

*Flavobacterium psychrophilum* is the causative agent of bacterial cold water disease and rainbow trout fry mortality syndrome in salmonid fishes and is associated with significant losses in the aquaculture industry. The virulence factors and molecular mechanisms of pathogenesis of *F. psychrophilum* are poorly understood. Moreover, at the present time, there are no effective vaccines and control using antimicrobial agents is problematic due to growing antimicrobial resistance and the fact that sick fish don’t eat. In the hopes of identifying vaccine and therapeutic targets, we sequenced the genome of the type strain ATCC 49418 which was isolated from the kidney of a Coho salmon (*Oncorhychus kisutch*) in Washington State (U.S.A.) in 1989. The genome is 2,715,909 bp with a G+C content of 32.75%. It contains 6 rRNA operons, 49 tRNA genes, and is predicted to encode 2,329 proteins.

## Introduction

*Flavobacterium psychrophilum* is a Gram-negative pathogen that infects all species of salmonid fish and has been found to also infect eel and three species of *cyprinids*[1-3]. It causes bacterial cold water disease (BCWD) and rainbow trout fry mortality syndrome (RTFS) in fish and is responsible for significant losses in the salmonid aquaculture industry [1]. Water temperature plays a key role in the infection and development of disease [4] which occurs between 4-16°C and is most prevalent at 10°C or below [5]. It was originally thought to be limited to North America [6] but it is now recognized in almost every country in Europe, in some parts of Asia, and in Australia [1,7].

Three serotypes and two biovars of *F. psychrophilum* have been described [7,8]. In addition, molecular analysis of the population structure of this bacterium suggests that there are a number of distinct lineages [7]. It has been speculated that some strains are species specific [9] while others are location specific [10]. Some strains have also been observed to cause only either BCWD or RTFS [7]. A recent study in Japan showed multiple sequence types infecting ayu (*Plecoglossus altivelis*) in a closed lake environment [11]. It is also known that phase variation can occur where the colonial phenotype changes between "rough" and "smooth", perhaps to help in evasion of the immune system [12]. Generally *F. psychrophilum* populations are heterogeneous; however, a recent study showed closely related epidemic clones infecting rainbow trout (*Oncorhynchus mykiss*) in Nordic countries [13]. To date, only one genome sequence [14] of *F. psychrophilum* has been reported and sequences of other strains are required to gain insight into the molecular mechanisms of virulence and why some strains are more virulent than others. Here we present a summary of classification and features of the *F. psychrophilum* type strain ATCC 49418 (= DSM 3660 = NCMB = 1947 = LMG 13179 = ATCC 49418) [15] together with a description of the complete genome and its annotation.

## Organism Information

### Classification and Features

The taxonomy of *F. psychrophilum* has been changed many times since Borg (1960) classified it as *Cytophaga psychrophila* based on its biochemical properties [16]. It was later reclassified within the genus *Flexibacter* based on DNA homology and renamed to *Flexibacter psychrophilus*[17]. Most recently, it was reclassified to the genus *Flavobacterium* and renamed to *F. psychrophilum* based on DNA-RNA hybridization [18]. The genus name was derived from the Latin *flavus* meaning "yellow" and the ancient Greek *βακτήριον* (baktḗrion) meaning "a small rod" giving the Neo-Latin word *Flavobacterium*, a "small yellow rod-shaped bacteria" [19,20]. The species name was derived from the Greek word *psuchros* (ψυχρός) meaning "cold" and the Neo-Latin word *philum* meaning "loving" which translates to "cold loving" [19,20]. The genus *Flavobacterium* consists of 119 recognized species [21]; it belongs to the family *Flavobacteriaceae*[18,22] and the order *Flavobacteriales*[23] (Table 1).

**Table 1:** **Classification and general features of***Flavobacterium psychrophilum***ATCC 49418**^**T**^

**MIGS ID**	**Property**	**Term**	**Evidence code**^**a**^
	Current Classification	Domain *Bacteria*	TAS [24]
		Phylum *Bacteroidetes*	TAS [25]
		Class *Flavobacteriia*	TAS [26,27]
		Order *Flavobacteriales*	TAS [23]
		Family *Flavobacteriacea*	TAS [18,22]
		Genus *Flavobacterium*	TAS [18,28]
		Species *Flavobacterium psychrophilum*	TAS [18]
		Type strain ATCC 49418	TAS [15,18]
	Gram stain	Negative	TAS [15]
	Cell shape	Rods	TAS [15]
	Motility	Gliding	TAS [15]
	Sporulation	Non-spore forming	TAS [18]
	Temperature range	Psychrotolerant (4°C to 30°C)	TAS [15,29,30]
	Optimum temperature	15-20°C	TAS [31,32]
	Carbon source	Non-saccharolytic	TAS [18]
	Energy source	Chemoorganotroph	TAS [18]
	Terminal electron receptor	Oxygen	NAS [33]
MIGS-6	Habitat	Host	TAS [15]
MIGS-6.3	Salinity	Usually grows in 0.5% and stops at 1.0%	TAS [8,15]
MIGS-22	Oxygen	Aerobic	TAS [15]
MIGS-15	Biotic relationship	Obligate pathogen of fish (but can survive in freshwater for several months)	NAS [7]
MIGS-14	Pathogenicity	Salmonid fishes, eel, and three species of *Cyprinids*	TAS [1,15]
MIGS-4	Geographic location	Worldwide including North America, Europe, and Asia	TAS [1,7]
MIGS-5	Sample collection time	1989	TAS [15]
MIGS-4.1 MIGS-4.2	Latitude – Longitude	Not reported	
MIGS-4.3	Depth	Not Reported	
MIGS-4.4	Altitude	Not Reported	

^a^Evidence codes - IDA: Inferred from Direct Assay; TAS: Traceable Author Statement (i.e., a direct report exists in the literature); NAS: Non-traceable Author Statement (i.e., not directly observed for the living, isolated sample, but based on a generally accepted property for the species, or anecdotal evidence). These evidence codes are from of the Gene Ontology project [40].

**Figure 1:**
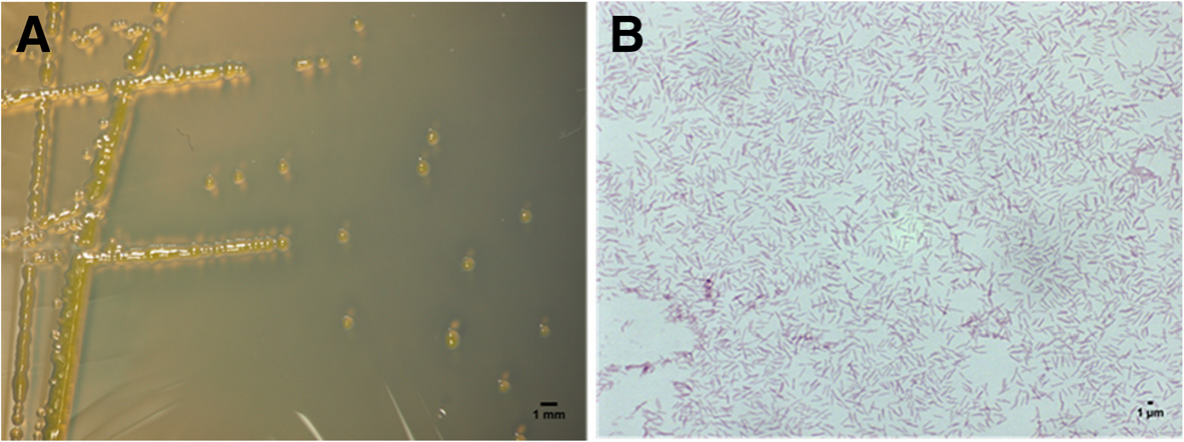
**Colonial and cellular morphology of *F. psychrophilum *** **ATCC 49418**^**T **^**grown on cytophaga agar (A) and Gram stained (B) (1000x).**

*F. psychrophilum* ATCC 49418^T^ was isolated in Washington State (U.S.A) from the kidney of a young Coho salmon (*Oncorhynchus kisutch*) in 1989 [15]. It is a Gram negative, aerobic, and psychrotolerant microorganism [7] (Figure 1). When grown on cytophaga agar, bright yellow, smooth, discreet, circular, convex, and non-adherent colonies are produced [8]. The optimal growth temperature is between 15-20°C [31,32] with no growth occurring at 30°C or greater [15,29,30]. Microscopically it is rod-shaped measuring 3–7 μm long and 0.3-0.5 μm wide [8]. Although gliding motility has been reported the mechanism is yet to be elucidated since *F. psychrophilum* does not appear to use pili or polysaccharide secretion [1,15,17]. API-ZYM tests show that it can produce alkaline phosphatase, esterase, lipase, leucine, valine, and cysteine arylamidases, trypsin, acid phosphatase, and napthol-AS-BI phosphohydrolase [8]. In addition, it has been reported that it can produce catalase [29,34] and oxidase [17], hydrolyze tributyrin and proteins including casein, gelatin, elastin, albumin, collagen, and fibrinogen [35-39]. Although many strains including ATTC 49418^T^ cannot metabolize simple and complex sugars [1] a recent study has shown that some strains are able to produce two or more sugar degrading enzymes including alpha-galactosidase, beta-galactosidase, alpha-glucosidase, beta-glucosidase, and N-acetyl-beta-glucosaminidase [8].

**Figure 2:**
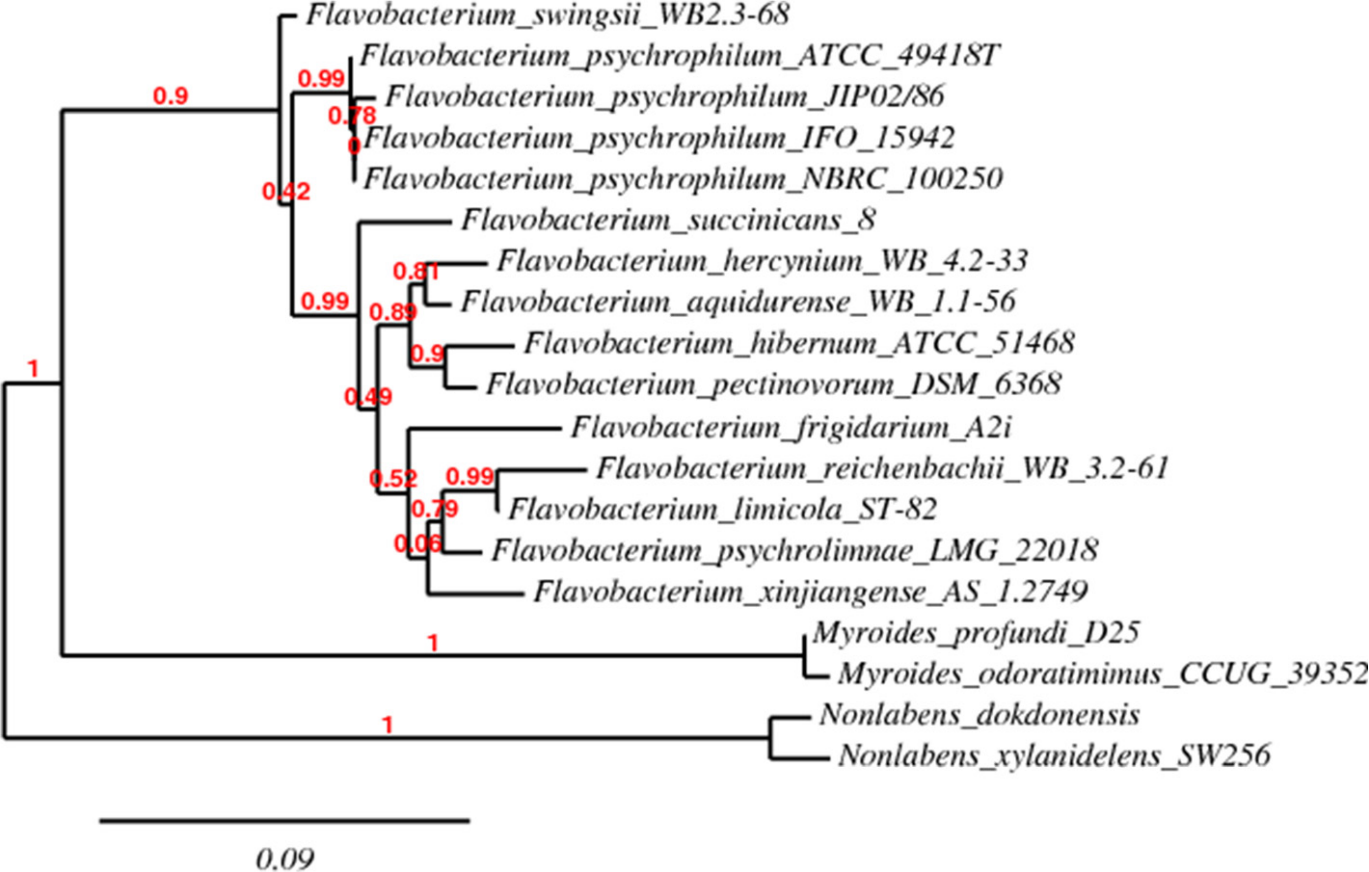
**Phylogenetic tree displaying the relationship between *F. psychrophilum *ATCC 49418**^**T **^**and selected strains and species of the same genus.** Other genera from the family *Flavobacteriaceae* were used as an out group. The phylogenetic tree was constructed using the "One Click" mode with default settings in the Phylogeny.fr platform [41]. This pipeline uses four different programs including MUSCLE [42], Gblock [43], PhyML [44], and TreeDyn [45]. The numbers above the branches are tree support values generated by PhyML using the aLRT statistical test.

A phylogentic tree was constructed using the 16S rRNA sequences of *F. psychrophilum* ATCC 49418^T^, selected strains and species of the same genus, as well as selected species of other genera belonging to the family *Flavobacteriaceae* (Figure 2). The four *F. psychrophilum* strains are grouped together in the tree with ATCC 49418^T^ being most similar to JIP02/86 (ATCC 49511), the only other strain to have a complete genome sequence.

## Genome sequencing information

### Genome project history

The complete genome sequence and annotation data of *F. psychrophilum* ATCC 49418^T^ have been deposited in DDBJ/EMBL/GenBank under the accession number CP007207. Sequencing and assembly steps as well as finishing were performed at McGill University and Génome Québec Innovation Centre. Annotation was performed using the NCBI Prokaryotic Genome Annotation Pipeline [46] and manually edited in Kodon (Applied Maths, Austin, TX). Table 2 presents a summary of the project information and its association with MIGS version 2.0 compliance [47].

**Table 2:** Project information

**MIGS ID**	**Property**	**Term**
MIGS-31	Finishing quality	Finished
MIGS-28	Libraries used	None
MIGS-29	Sequencing platforms	PacBio RS II
MIGS-31.2	Fold coverage	184x
MIGS-30	Assemblers	HGAP workflow
MIGS-32	Gene calling method	NCBI Prokaryotic Genome Annotation Pipeline, GeneMarkS+
	Locus Tag	FPG3
	GenBank ID	CP007207
	GenBank Date of Release	September 12, 2014
	BioProject ID	PRJNA236029
	GOLD ID	Gi0074339
	Project relevance	Fish Pathogen
MIGS-13	Source Material Identifier	ATCC

### Growth conditions and DNA isolation

*F. psychrophilum* ATCC 49418^T^ was originally obtained from the American Type Culture Collection [15] and was stored in a frozen glycerol stock (15%) at -70°C. It was grown for 4 days at 12°C on modified cytophaga agar [48] containing 0.06% (*w/v*) tryptone, 0.05% yeast extract, 0.02% beef extract, 0.02% sodium acetate, 0.05% anhydrous calcium chloride, 0.05% magnesium chloride, 0.05% potassium chloride, 1.5% agar, 0.02% gelatin, pH 7.5. Well isolated colonies were used for genomic DNA isolation. Colonies (~ 4 mm^3^) were picked using a sterile toothpick and lysed using modified B1 (1 50 mM Tris·Cl, 50 mM EDTA, 0.5% Tween®-20, 0.5% Triton X-100, pH 8.0) and B2 (750 mM NaCl, 50 mM MOPS, 15% isopropanol, 0.15% Triton X-100, pH 7.0) buffers. DNA was purified and eluted using the QIAGEN Plasmid Midi Kit (Qiagen, Germany) following manufacturer's protocol.

### Genome sequencing and assembly

Genome sequencing of *F. psychrophilum* ATCC 49418^T^ was performed using a PacBio RS II instrument. The reads were automatically processed through the Single Molecule Real Time (SMRT) software suite using the Hierarchical Genome Assembly Processing (HGAP) pipeline [49]. The resulting reads (580,625,890 bp in total) were filtered and the longest reads with 20x coverage were selected as seeds for constructing preassemblies. The preassemblies were constructed by aligning the short reads to the long reads (seeds). Each read was mapped to multiple seeds using BLASTR [50]. In total there were 8073 long sequences totaling 90,000,401 bp with an average length of 11148 bp and 162,858 bp short sequences totaling 490,625,489 bp with an average length of 3013 bp. Since errors in PacBio are random, aligning the multiple short reads onto the long reads allows the correction of errors in the long reads. The optimal number of sequences to be mapped onto the seeds is controlled by the "-bestn" parameter and the optimal number was determined to be 12. The preassembled reads for the seeds are generated using PBDAG-Con [51] to create corrected consensus sequences in addition to quality analysis of the seeds. This script uses multiple sequence alignments and a directed acyclic graph to produce the best consensus reads possible. It does so by eliminating the insertion and deletion errors generated during the sequencing process. In addition, it avoids generating chimeric sequences (sequences with artifacts) for assembly because chimeric reads will have no or low short sequence coverage. At the end of the process, only the best preassembled reads without artifacts are sent to the assembler [52].

After quality analysis and eliminating some of the preassembled reads by PBDAG-Con, the remaining 6,009 reads were fed into the Celera assembler which uses an overlap-layout-consensus strategy [49]. A total of 2 contigs were generated with sizes 1,647,861 bp and 1,076,634 bp. These contigs underwent an additional polishing step where they were compared against the raw reads and any artifacts found were removed [49]. The final consensus generated was analyzed and improved by using the multiread consensus algorithm Quiver. Quiver takes the two contigs and the initial sequencing reads and maps the reads onto the assemblies [49]. It then disregards the alignment between the reads and the assemblies and a consensus is created independently from the reads allowing it to remove any fine-scale errors made by the Celera assembler [52]. An approximate copy of the consensus sequences is then generated by Quiver which makes insertions and deletions and those that improve the maximum likelihood are applied to the initial consensus sequence [53]. The two final contigs generated by Quiver were 1,648,613 bp and 1,077,094 bp.

The two contigs underwent a finishing process using SeqMan Pro (DNASTAR Inc., Madison, WI). The two contigs were collapsed into one and the sequence was then opened in a region homologous to the Ori of *F. psychrophilum* JIP02/86 resulting in another two contigs. These were resealed using SeqMan Pro to create one final complete contig.

### Genome annotation

The NCBI Prokaryotic Genome Annotation Pipeline was used to predict protein coding genes, structural RNAs (5S, 16S, 23S), tRNAs, and small non-coding RNAs [54]. Protein coding genes were predicted by protein alignment using ProSplign [55] where only complete alignments with 100% identity to a reference protein are kept for final annotation. Frameshifted or partial alignments were further analyzed by GeneMarkS+ [56] for further analysis and gene prediction. A BLASTN search against a reference set of structural RNA genomes from the NCBI Reference Sequence Collection was conducted to find the structural RNAs since they are highly conserved in closely related prokaryotes. tRNAscan-SE was used to identify the tRNAs [57]. Small RNAs were predicted using a BLASTN search against sequences of selected Rfam families and the results were refined further using Cmsearch [58]. Clustered Regularly Interspaced Short Palindromic Repeats (CRISPRs) were identified by searching the CRISPR database with the CRISPRfinder program (http://crispr.u-psud.fr/Server/) [59-62].

## Genome properties

**Figure 3:**
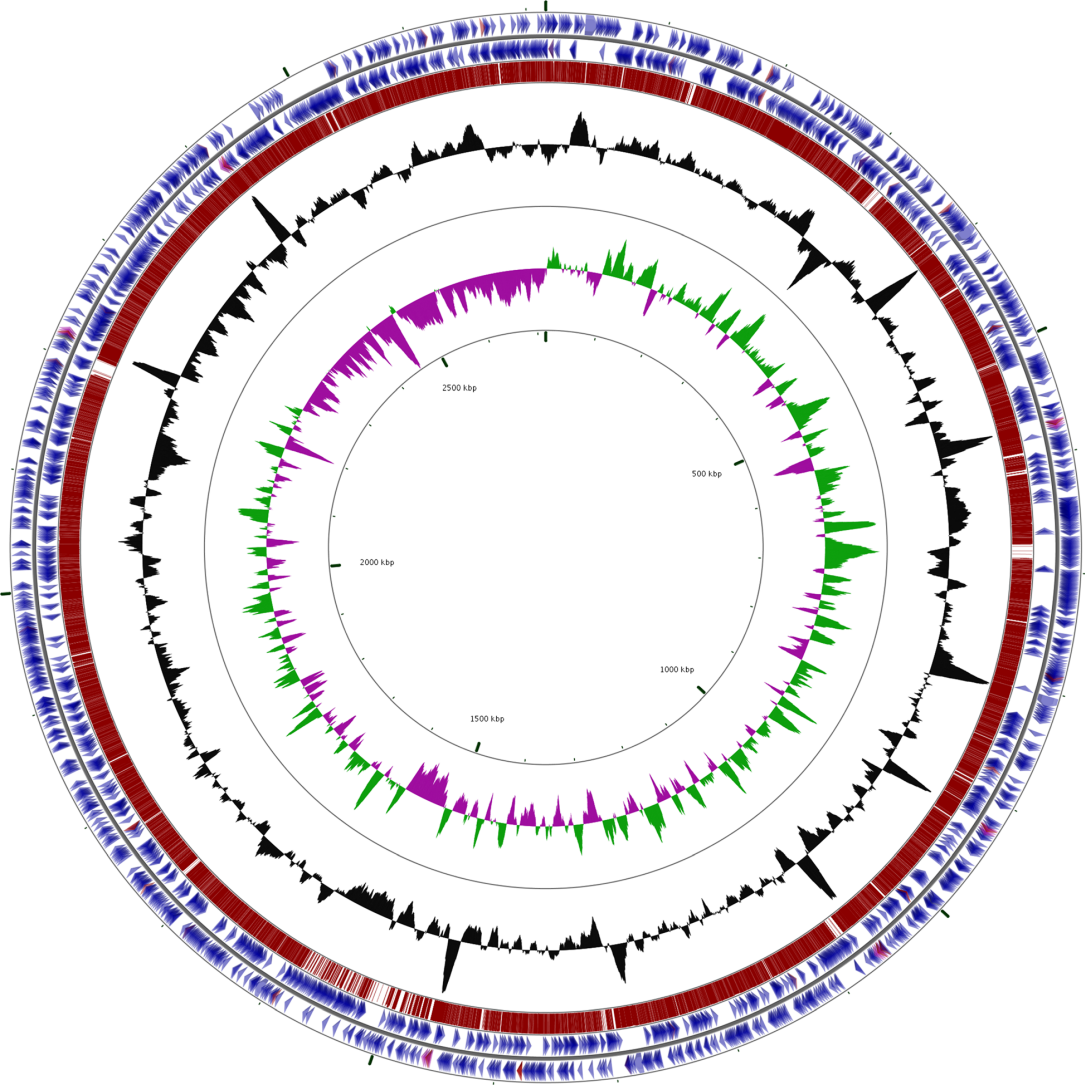
**Comparison of *F. psychrophilum *ATCC 49418**^**T **^**and *F. psychrophilum *JIP02/86 (NC_009613.3) created using CGview [**[65]**].** From the outside to the center: Genes on forward strand (blue clockwise arrows), genes on reverse strand (blue counter-clockwise arrows), *F. psychrophilum* JIP02/86 genome (red), RNA genes (tRNAs orange, rRNAs violet, other RNAs gray), GC content (black), GC skew (purple/olive).

The 2,715,909 bp (32.75% G+C) genome of *F. psychrophilum* ATCC 49418^T^ contains 6 rRNA operons and 49 tRNA genes and is predicted to encode 2329 proteins (Figure 3 and Table 3). No plasmids were identified during the annotation process. The distribution of genes into COG functions is shown in Table 4. When compared to the JIP02/86 strain, ATCC 49418^T^ had fewer proteins classified as “not in COGs” (43.8% vs. 47.5%) and had slightly more replication, repair, and recombination COGs (96 vs. 82). The two strains differed little in other COG categories. The Average Nucleotide Identity (ANI) between ATCC 49418^T^ and JIP02/86 was calculated to be 99.34% (+/-1.83%) and 99.37% (+/- 1.71%) one way and 99.43% (+/- 1.51%) two way [63]. The estimated distance to distance hybridization (DDH) values between the two strains was calculated to be 96.20% (+/- 1.16%) and the distance was 0.0053. The probability that DDH>70% (i.e. same species) is 97.48% [64].

**Table 3:** Nucleotide content and gene count levels of the genome

**Attribute**	**Genome (total)**	
	**Value**	**% of total**
Genome size (bp)	2,715,909	100.00%
DNA coding (bp)	2,336,075	86.01%
G+C content (bp)	889,460	32.75%
DNA scaffolds	1	
Total genes	2397	100.00%
Protein-coding genes	2,329	97.00%
RNA genes	68	2.84%
Pseudo genes	24	1.00%
Genes in internal clusters	N/D^a^	
Genes with function prediction	1881	78.47%
Genes assigned to COGs	1,438	60.00%
Genes assigned Pfam domains	1,933	80.64%
Genes with signal peptides	236	9.85%
Genes with transmembrane helices	506	21.11%
Number of CRISPR candidates	8	
Confirmed CRISPR(s)	1	
Unconfirmed CRISPR(s)	7	

^a^N/D = not determined.

**Table 4:** Number of genes associated with the 25 general COG functional categories

**Code**	**Value**	**% of total**	**Description**
J	140.0	9.66	Translation
A	0.0	0.00	RNA processing and modification
K	72.0	4.97	Transcription
L	96.0	6.63	Replication, recombination and repair
B	0.0	0.00	Chromatin structure and dynamics
D	18.0	1.24	Cell cycle control, mitosis and meiosis
Y	0.0	0.00	Nuclear structure
V	41.0	2.83	Defense mechanisms
T	32.0	2.21	Signal transduction mechanisms
M	145.0	10.01	Cell wall/membrane biogenesis
N	4.0	0.28	Cell motility
Z	1.0	0.07	Cytoskeleton
W	0.0	0.00	Extracellular structures
U	31.0	2.14	Intracellular trafficking and secretion
O	61.0	4.21	Posttranslational modification, protein turnover, chaperones
C	75.0	5.16	Energy production and conversion
G	51.0	3.52	Carbohydrate transport and metabolism
E	120.0	8.28	Amino acid transport and metabolism
F	54.0	3.73	Nucleotide transport and metabolism
H	96.0	6.63	Coenzyme transport and metabolism
I	63.0	4.35	Lipid transport and metabolism
P	70.0	4.83	Inorganic ion transport and metabolism
Q	26.0	1.79	Secondary metabolites biosynthesis, transport and catabolism
R	164.0	11.32	General function prediction only
S	89.0	6.14	Function unknown
-	1050	43.80	Not in COGs

## Insights into the genome sequence

A number of studies have been done to determine the pathogenesis of *F. psychrophilum* but, to date, the exact mechanisms are still unknown [1]. Some putative and previously characterized virulence factors are listed in Table 5. Proteolytic enzymes are widely used by fish pathogens to cause tissue damage and allow invasion of the host [1]. In the *F. psychrophilum* ATCC 49418^T^ genome there are four metalloprotease encoding genes including a predicted zinc metalloprotease [FPG3_00455], a predicted zinc peptidase [FPG3_06120] and the previously reported Fpp1 [66] and Fpp2 [67] metalloproteases. Rainbow trout with RTFS are anemic and past studies have reported that the red blood cells of rainbow trout are partially lysed when infected by *F. psychrophilum*[68,69]. Homologs of two RTX hemolysin transporters (FPG3_06485, FPG3_10400) were identified, but did not appear to be linked to any toxin or modification genes [70]. Six iron transport genes were also identified; these were anticipated since iron uptake is a well-known characteristic of most pathogens. Moreover, recent research has shown that attenuated *F. psychrophilum* strains cultured under iron limiting conditions confer greater protection to fish when used as an experimental vaccine [71]. A hydroperoxidase with predicted catalase and peroxidase functions were also identified. In addition, there are 11 cell surface proteins with leucine rich repeats that are predicted to be adhesins; several are listed in Table 5. These were very similar to the ones found in *F. psychrophilum* JIP/02. Further research is required to determine what functions these adhesins have and how they help *F. psychrophilum* bind to the host.

**Table 5:** **Some putative virulence factors of *F. psychrophilum *** **ATCC 49418**

**Locus tag**	**Gene name**	**Family**	**Product**
FPG3_00455		M50	Putative zinc metalloprotease
FPG3_01260	*fpp1*	M12B	Psychrophilic metalloprotease Fpp1 precursor
FPG3_01265	*fpp2*	M43	Psychrophilic metalloprotease Fpp2 precursor
FPG3_06120		Zn Peptidase	Putative neutral zinc metallopeptidase
FPG3_06485	*hlyD*	HlyD2	Putative hemolysin D transporter
FPG3_10400	*hlyD*	HlyD2	Putative hemolysin D transmembrane transporter
FPG3_00420		MntH	Mn^2+^ and Fe^2+^ transporter of the NRAMP family
FPG3_00490		FeoA	Iron transport protein A
FPG3_00495		FeoB	Iron transport protein B
FPG3_04340		Peptidase M75	Iron-regulated protein A precursor
FPG3_04455		TM-ABC Iron Siderophore	ABC iron transporter system, permease component
FPG3_05120		FeoB	ABC iron transporter system, binding protein precursor
FPG3_06195		CCC1	Probable iron transporter
FPG3_09395		Plant peroxidase like	Hydroperoxidase with catalase and peroxidase activities
FPG3_00925		LRR5	Cell surface protein precursor with leucine rich repeats
FPG3_00930		LRR5	Cell surface protein precursor with leucine rich repeats
FPG3_00935		LRR5	Cell surface protein precursor with leucine rich repeats
FPG3_00940		LRR5	Cell surface protein precursor with leucine rich repeats

## Conclusion

*Flavobacterium psychrophilum*, the causative agent of BCWD and RTFS in salmonid fishes, causes significant economic losses in the aquaculture industry. The genome sequence of the ATCC 49418^T^ strain will hopefully provide new insights into virulence mechanisms and pathogenesis of *F. psychrophilum* and help in the identification of suitable targets for vaccines and antimicrobial agents; however, to do this much more analysis will be required.

## Abbreviations

BCWD: Bacterial cold water disease; RTFS: Rainbow trout fry mortality syndrome.

## Competing interests

The authors declare that they have no competing interests.

## Authors’ contributions

AW participated in genome sequencing analysis, bioinformatics analysis, drafted the original manuscript, and participated in the revision process. AK participated in genome sequence analysis and assembly refinement. JL and BD participated in the study design and provided funding for the project. JM conceived the study, provided funding for the project, and participated in the revision process. All authors read and approved the final manuscript.

## References

[B1] Nematollahi A Decostere A Pasmans F Haesebrouck F *Flavobacterium psychrophilum* infections in salmonid fish J 2003 26 10 563 74 http://dx.doi.org/10.1046/j.1365-2761.2003.00488.x 10.1046/j.1365-2761.2003.00488.x 14653314

[B2] Austin B Austin DA Bacterial fish pathogens : Disease of farmed and wild fish 1999 Third Chichester: Praxis Publishing

[B3] Lehmann J Mock D Stiirenberg F Bernardet J First isolation of *Cytophaga psychrophila* from a systemic disease in eel and cyprinids Dis 1991 10 217 20 http://dx.doi.org/10.3354/dao010217

[B4] Noga EJ Fish disease: Diagnosis and treatment 2010 Ames, Iowa: Wiley-Blackwell

[B5] Holt RA *Cytophaga psychrophila*, the causative agent of bacterialcold water disease in salmonid fish PhD thesis 1987 Oregon, USA: University of Oregon

[B6] Anderson JI Conroy DA The pathogenic myxobacteria with special reference to fish diseases J 1969 32 1 30 9 10.1111/j.1365-2672.1969.tb02186.x 5815479

[B7] Barnes ME Brown ML A review of *Flavobacterium psycrhophilum* biology, clinical, signs, and bacterial cold water disease prevention and treatment Open Fish Sci 2011 4 1 9 10.2174/1874401X01104010001

[B8] Hesami S Allen KJ Metcalf D Ostland VE MacInnes JI Lumsden JS Phenotypic and genotypic analysis of *Flavobacterium psychrophilum* isolates from ontario salmonids with bacterial coldwater disease Can 2008 54 8 619 29 10.1139/W08-058 18772924

[B9] Nagai T Nakai T Growth of *Flavobacterium psychrophilum* in fish serum correlates with pathogenicity J 2011 34 4 303 10 http://dx.doi.org/10.1111/j.1365-2761.2011.01245.x 10.1111/j.1365-2761.2011.01245.x 21382051

[B10] Chakroun C Grimont F Urdaci MC Bernardet JF Fingerprinting of *Flavobacterium psychrophilum* isolates by ribotyping and plasmid profiling Dis 1998 33 3 167 77 9841121 10.3354/dao033167

[B11] Nagata EF Darmon CC Bernardet JF Eguchi M Duchaud E Nicolas P Population structure of the fish pathogen *Flavobacterium psychrophilum* at whole-country and model river levels in Japan Vet 2013 44 34 42 10.1186/1297-9716-44-34 23682575 PMC3660162

[B12] Sundell K Heinikainen S Wiklund T Structure of *Flavobacterium psychrophilum* populations infecting farmed rainbow trout *Oncorhynchus mykiss* Dis 2013 103 2 111 19 10.3354/dao02573 23548361

[B13] Nilsen H Sundell K Duchaud E Nicolas P Dalsgaard I Madsen L Aspan A Jansson E Colquhoun DI Wiklund T Multilocus sequence typing identifies epidemic clones of *Flavobacterium psychrophilum* in nordic countries Appl 2014 80 9 2728 36 10.1128/AEM.04233-13 24561585 PMC3993282

[B14] Duchaud E Boussaha M Loux V Bernardet J Michel C Kerouault B Mondot S Nicolas P Bossy R Caron C Bessières P Gibrat JF Claverol S Dumetz F Le Hénaff M Benmansour A Complete genome sequence of the fish pathogen *Flavobacterium psychrophilum* Nat 2007 25 7 763 69 http://dx.doi.org/10.1038/nbt1313 10.1038/nbt1313 17592475

[B15] Bernardet JF Kerouault B Phenotypic and genomic studies of "*Cytophaga psychrophila*" isolated from diseased rainbow trout (oncorhynchus mykiss) in France Appl 1989 55 7 1796 1800 2764577 10.1128/aem.55.7.1796-1800.1989 PMC202952

[B16] Borg AF Studies on Myxobacteria associated with diseases in salmonid fishes 1960 8 Washington, D.C: American Association for the Advancement of Science

[B17] Bernardet JF Grimont PAD Deoxyribonucleic acid relatedness and phenotypic characterization of *Flexibacter columnaris* sp. nov., nom. rev., *Flexibacter psychrophilus* sp. nov., nom. rev., and *Flexibacter maritimus* wakabayashi, hikida, and masumura Int 1989 39 3 346 54 http://dx.doi.org/10.1099/00207713-39-3-346 10.1099/00207713-39-3-346

[B18] Bernardet JF Segers P Vancanneyt M Berthe F Kersters K Vandamme P Cutting a gordian knot: Emended classification and description of the genus *Flavobacterium*, emended description of the family *Flavobacteriaceae*, and proposal of *Flavobacterium hydatis* nom. nov. (basonym, *Cytophaga aquatilis* strohl and tait 1978) Int 1996 46 1 128 48 http://dx.doi.org/10.1099/00207713-46-1-128 10.1099/00207713-46-1-128

[B19] Euzeby JP List of bacterial names with standing in nomenclature: A folder available on the internet Int 1997 47 2 590 92 10.1099/00207713-47-2-590 9103655

[B20] Schlegel HG General microbiology 1993 7 Cambridge, U.K: Cambridge University Press

[B21] Parker CT Wigley S Garrity GM Parker CT, Garrity GM Taxonomic Abstract for the genus Flavobacterium The NamesforLife Abstracts [Internet]. NamesforLife, LLC Available from: http://dx.doi.org/10.1601/tx.8071

[B22] Bernardet JF Krieg NR, Staley JT, Brown DR, Hedlund BP, Paster BJ, Ward NL, Ludwig W, Whitman WB Family I. *Flavobacteriaceae* reichenbach 1992b, 327VP (effective publication: Reichenbach 1989b, 2013.) emend. Bernardet, Segers, Vancanneyt, Berthe, Kersters and Vandamme 1996, 145 emend. Bernardet, Nakagawa and Holmes 2002, 1057 Bergey's manual of systematic bacteriology 2010 4 Second New York: Springer 106

[B23] Bernardet JF Krieg NR, Staley JT, Brown DR, Hedlund BP, Paster BJ, Ward NL, Ludwig W, Whitman WB Order I. *Flavobacteriales* ord. nov Bergey's manual of systematic bacteriology 2010 4 Second New York: Springer 105

[B24] Woese CR Kandler O Wheelis ML Towards a natural system of organisms: Proposal for the domains *Archaea, bacteria*, and *Eucarya* Proc 1990 87 12 4576 79 http://www.ncbi.nlm.nih.gov/pmc/articles/PMC54159/10.1073/pnas.87.12.4576 2112744 PMC54159

[B25] Krieg NR Ludwig W Euzéby J Whitman WB Krieg NR, Staley JT, Brown DR, Hedlund BP, Paster BJ, Ward NL, Ludwig W, Whitman WB Phylum XIV. *Bacteroidetes* phyl. nov Bergey's manual of systematic bacteriology 2010 4 Second New York: Springer 25

[B26] Validation list no. 145. list of new names and new combinations previously effectively, but not validly, published Int 2012 62 1017 19 http://ijs.sgmjournals.org/content/62/Pt_5/1017.full

[B27] Bernardet JF Krieg NR, Staley JT, Brown DR, Hedlund BP, Paster BJ, Ward NL, Ludwig W, Whitman WB Class II. *Flavobacteriia* class. nov Bergey's manual of systematic bacteriology 2010 4 Second New York: Springer 105

[B28] Reichenbach H Staley JT, Bryant MP, Pfennig N, Holt JG *Genus 1. Cytophaga* Winogradsky *1929, 577*^*AL*^*, emend* Bergey's manual of systematic bacteriology 1989 3 Baltimore: The Williams & Wilkins Co 2015 50

[B29] Pacha RE Characteristics of *Cytophaga psychrophila* (borg) isolated during outbreaks of bacterial cold-water disease Appl 1968 16 1 97 101 4169926 10.1128/am.16.1.97-101.1968 PMC547320

[B30] Cepeda C Garci´a-Márquez S Santos Y Improved growth of *Flavobacterium psychrophilum* using a new culture medium Aquaculture 2004 238 1–4 75 82 http://dx.doi.org/10.1016/j.aquaculture.2004.05.013

[B31] Holt RA Amandi A Rohovec JS Fryer JL Relation of water temperature to bacterial cold water disease in coho salmon, chinook salmon, rainbow trout J. Aquat. Anim. Health 1989 1 94 101 10.1577/1548-8667(1989)001<0094:ROWTTB>2.3.CO;2

[B32] Uddin MN Wakabayashi H Effects of temperature on growth and protease production of, Cytophaga psvchrophila Fish Pathol 1997 32 4 225 26 10.3147/jsfp.32.225

[B33] Bernardet JF Bowman JP Dworkin M, Falkow S, Rosenberg E, Schleifer K The genus *Flavobacterium* The Prokaryotes. A handbook on the biology of bacteria: Proteobacteria: Delta and epsilon subclasses. deeply rooting bacteria 2006 7 3 New York: Springer 486 509

[B34] Pacha RE Porter S Characteristics of myxobacteria isolated from the surface of freshwater fish Appl 1969 16 12 1901 06 10.1128/am.16.12.1901-1906.1968 PMC547793 16349828

[B35] Bertolini JM Wakabayashi H Watral VG Whipple MJ Rohovec JS Electrophoretic detection of proteases from selected strains of *Flexibacter psychrophilus* and assessment of their variability J.Aquat.Anim.Health 1994 6 3 224 33 http://www.tandfonline.com/doi/abs/10.1577/1548-8667%281994%29006%3C0224%3AEDOPFS%3E2.3.CO%3B2?ai=t8bz&mi=bkerqn&af=R 10.1577/1548-8667(1994)006<0224:EDOPFS>2.3.CO;2

[B36] Ostland VE Byrne PJ Hoover G Ferguson HW Necrotic myositis of rainbow trout,*Oncorhynchus mykiss* (walbaum): Proteolytic characteristics of a crude extracellular preparation from *Flavobacterium psychrophilum* J 2000 23 5 329 36 http://dx.doi.org/10.1046/j.1365-2761.2000.00251.x 10.1046/j.1365-2761.2000.00251.x

[B37] Moller JD Ellis AE Barnes AC Dalsgaard I Iron acquisition mechanisms of *Flavobacterium psychrophilum* J 2005 28 7 391 98 http://dx.doi.org/10.1111/j.1365-2761.2005.00639.x 10.1111/j.1365-2761.2005.00639.x 16083444

[B38] Otis EJ Lesions of cold water disease in steelhead trout (Salmo gairdneri): The role of Cytophaga psychrophila extracellular products 1984 MSc thesis: University of Rhode Island

[B39] Holt RA Rohovec JS Fryer JL Inglis V, Roberts RJ, Bromage NR Bacterial cold-water disease Bacterial diseases of fish 1993 Oxford: Blackwell Scientific Publications 3 23

[B40] Ashburner M Ball CA Blake JA Botstein D Butler H Cherry JM Davis AP Dolinski K Dwight SS Eppig JT Harris MA Hill DP Issel-Tarver L Kasarskis A Lewis S Matese JC Richardson JE Ringwald M Rubin GM Sherlock G Gene ontology: Tool for the unification of biology. the gene ontology consortium Nat 2000 25 1 25 9 10.1038/75556 10802651 PMC3037419

[B41] Dereeper A Guignon V Blanc G Audic S Buffet S Chevenet F Dufayard JF Guindon S Lefort V Lescot M Claverie JM Gascuel O Phylogeny.fr: Robust phylogenetic analysis for the non-specialist Nucleic Acids Res 2008 36 Web Server issue W465-9 18424797 10.1093/nar/gkn180 PMC2447785

[B42] Edgar RC MUSCLE: Multiple sequence alignment with high accuracy and high throughput Nucleic Acids Res 2004 32 5 1792 97 http://dx.doi.org/10.1093/nar/gkh340 10.1093/nar/gkh340 15034147 PMC390337

[B43] Castresana J Selection of conserved blocks from multiple alignments for their use in phylogenetic analysis Mol 2000 17 4 540 52 10.1093/oxfordjournals.molbev.a026334 10742046

[B44] Guindon S Gascuel O A simple, fast, and accurate algorithm to estimate large phylogenies by maximum likelihood Syst 2003 52 5 696 704 http://dx.doi.org/10.1080/10635150390235520 10.1080/10635150390235520 14530136

[B45] Chevenet F Brun C Banuls AL Jacq B Christen R TreeDyn: Towards dynamic graphics and annotations for analyses of trees BMC Bioinformatics 2006 7 439 10.1186/1471-2105-7-439 17032440 PMC1615880

[B46] NCBI Prokaryotic Genome Annotation Pipelinehttp://www.ncbi.nlm.nih.gov/genome/annotation_prok/10.1093/nar/gkw569 PMC5001611 27342282

[B47] Field D Garrity G Gray T Morrison N Selengut J Sterk P Tatusova T Thomson N Allen MJ Angiuoli SV Ashburner M Axelrod N Baldauf S Ballard S Boore J Cochrane G Cole J Dawyndt P De Vos P dePamphilis C Edwards R Faruque N Feldman R Gilbert J Gilna P Glockner FO Goldtein P Guralnick R Half D Hancock D The minimum information about a genome sequence (MIGS) specification Nat 2008 26 5 541 47 10.1038/nbt1360 18464787 PMC2409278

[B48] Anacker RL Ordal EJ Studies on the myxobacterium *Chondrococcus columnaris.* I. serological typing J 1959 78 1 25 32 13672906 10.1128/jb.78.1.25-32.1959 PMC290480

[B49] Chin CS Alexander DH Marks P Klammer AA Drake J Heiner C Clum A Copeland A Huddleston J Eichler EE Turner SW Korlach J Nonhybrid, finished microbial genome assemblies from long-read SMRT sequencing data Nat. Methods 2013 10 6 563 69 10.1038/nmeth.2474 23644548

[B50] Chaisson MJ Tesler G Mapping single molecule sequencing reads using basic local alignment with successive refinement (BLASR): Application and theory BMC Bioinformatics 2012 13 238-2105-13-238 22988817 10.1186/1471-2105-13-238 PMC3572422

[B51] PBDAG-Con https://github.com/PacificBiosciences/pbdagcon

[B52] Chain PS Grafham DV Fulton RS Fitzgerald MG Hostetler J Muzny D Ali J Birren B Bruce DC Buhay C Cole JR Ding Y Dugan S Field D Garrity GM Gibbs R Graves T Han CS Harrison SH Highlander S Hugenholtz P Khouri HM Kodira CD Kolker E Kyrpides NC Lang D Lapidus A Malfatti SA Markowitz V Metha T Genomics. genome project standards in a new era of sequencing Science 2009 326 5950 236 37 10.1126/science.1180614 19815760 PMC3854948

[B53] Lee C Grasso C Sharlow MF Multiple sequence alignment using partial order graphs Bioinformatics 2002 18 3 452 64 http://dx.doi.org/10.1093/bioinformatics/18.3.452 10.1093/bioinformatics/18.3.452 11934745

[B54] Angiuoli SV Gussman A Klimke W Cochrane G Field D Garrity G Kodira CD Kyrpides N Madupu R Markowitz V Tatusova T Thomson N White O Toward an online repository of standard operating procedures (SOPs) for (meta)genomic annotation OMICS 2008 12 2 137 41 10.1089/omi.2008.0017 18416670 PMC3196215

[B55] Wheeler DL Database resources of the national center for biotechnology information Nucleic Acids Res 2006 34 90001 173 80 http://dx.doi.org/10.1093/nar/gkj158 10.1093/nar/gkj158 PMC1347520 16381840

[B56] Besemer J Lomsadze A Borodovsky M GeneMarkS: A self-training method for prediction of gene starts in microbial genomes. implications for finding sequence motifs in regulatory regions Nucleic Acids Res 2001 29 12 2607 18 10.1093/nar/29.12.2607 11410670 PMC55746

[B57] Lowe TM Eddy SR tRNAscan-SE A program for improved detection of transfer RNA genes in genomic sequence Nucleic Acids Res 1997 25 5 955 64 http://dx.doi.org/10.1093/nar/25.5.0955 10.1093/nar/25.5.0955 9023104 PMC146525

[B58] Griffiths-Jones S Rfam An RNA family database Nucleic Acids Res 2003 31 1 439 41 http://dx.doi.org/10.1093/nar/gkg006 10.1093/nar/gkg006 12520045 PMC165453

[B59] Rousseau C Gonnet M Le Romancer M Nicolas J CRISPI: A CRISPR interactive database Bioinformatics 2009 25 24 3317 18 10.1093/bioinformatics/btp586 19846435 PMC2788928

[B60] Grissa I Vergnaud G Pourcel C The CRISPRdb database and tools to display CRISPRs and to generate dictionaries of spacers and repeats BMC Bioinformatics 2007 8 172 82 10.1186/1471-2105-8-172 17521438 PMC1892036

[B61] Grissa I Vergnaud G Pourcel C CRISPRFinder: A web tool to identify clustered regularly interspaced short palindromic repeats Nucleic Acids Res 2007 35 W52 7 10.1093/nar/gkm360 17537822 PMC1933234

[B62] Grissa I Vergnaud G Pourcel C CRISPRcompar: A website to compare clustered regularly interspaced short palindromic repeats Nucleic Acids Res 2008 36 W145 8 10.1093/nar/gkn228 18442988 PMC2447796

[B63] ANI Average Nucleotide Indexhttp://enve-omics.ce.gatech.edu/ani/index

[B64] Auch AF von Jan M Klenk HP Digital DNA-DNA hybridization for microbial species delineation by means of genome-to-genome sequence comparison Stand 2010 2 1 117 34 10.4056/sigs.531120 21304684 PMC3035253

[B65] Stothard P Wishart DS Circular genome visualization and exploration using CGView Bioinformatics 2004 21 4 537 39 http://dx.doi.org/10.1093/bioinformatics/bti054 15479716 10.1093/bioinformatics/bti054

[B66] Secades P Alvarez B Guijarro JA Purification and characterization of a psychrophilic, calcium-induced, growth-phase-dependent metalloprotease from the fish pathogen *Flavobacterium psychrophilum* Appl 2001 67 6 2436 44 10.1128/AEM.67.6.2436-2444.2001 11375148 PMC92892

[B67] Secades P Alvarez B Guijarro JA Purification and properties of a new psychrophilic metalloprotease (Fpp2) in the fish pathogen *Flavobacterium psychrophilum* FEMS Microbiol 2003 226 2 273 79 http://dx.doi.org/10.1016/s0378-1097(03)00599-8 10.1016/S0378-1097(03)00599-8 14553922

[B68] Lorenzen E Study on Flexibacter psychrophilus in relation to rainbow trout fry syndrome (RTFS). PhD thesis 1994 Copenhagen, Denmark: Royal Veterinary and Agricultural University

[B69] Lorenzen E Olesen N Characterization of isolates of *Flavobacterium psychrophilum* associated with coldwater disease or rainbow trout fry syndrome:Serological studies Dis 1997 31 209 20 http://dx.doi.org/10.3354/dao031209

[B70] Koronakis V Cross M Senior B Koronakis E Hughes C The secreted hemolysins of *Proteus mirabilis*, *Proteus vulgaris*, and *Morganella morganii* are genetically related to each other and to the alpha-hemolysin of *Escherichia coli* J 1987 169 4 1509 15 3549692 10.1128/jb.169.4.1509-1515.1987 PMC211976

[B71] Long A Fehringer TR Swain MA LaFrentz BR Call DR Cain KD Enhanced efficacy of an attenuated *Flavobacterium psychrophilum* strain cultured under iron-limited conditions Fish Shellfish Immunol 2013 35 5 1477 82 10.1016/j.fsi.2013.08.009 23989039

